# Multiple Visual Field Representations in the Visual Wulst of a Laterally Eyed Bird, the Zebra Finch (*Taeniopygia guttata*)

**DOI:** 10.1371/journal.pone.0154927

**Published:** 2016-05-03

**Authors:** Hans-Joachim Bischof, Dennis Eckmeier, Nina Keary, Siegrid Löwel, Uwe Mayer, Neethu Michael

**Affiliations:** 1 Verhaltensforschung, Universität Bielefeld, Bielefeld, Germany; 2 Champalimaud Neuroscience Programme, Center for the Unknown, Lisbon, Portugal; 3 Department of Systems Neuroscience, Johann-Friedrich-Blumenbach Institut für Zoologie und Anthropologie, Universität Göttingen, Göttingen, Germany; 4 Center for Mind/Brain Sciences, University of Trento, Rovereto, Italy; 5 Göttingen Graduate School for Neurosciences, Biophysics, and Molecular Biosciences (GGNB), Göttingen, Germany; Utrecht University, NETHERLANDS

## Abstract

The visual wulst is the telencephalic target of the avian thalamofugal visual system. It contains several retinotopically organised representations of the contralateral visual field. We used optical imaging of intrinsic signals, electrophysiological recordings, and retrograde tracing with two fluorescent tracers to evaluate properties of these representations in the zebra finch, a songbird with laterally placed eyes. Our experiments revealed that there is some variability of the neuronal maps between individuals and also concerning the number of detectable maps. It was nonetheless possible to identify three different maps, a posterolateral, a posteromedial, and an anterior one, which were quite constant in their relation to each other. The posterolateral map was in contrast to the two others constantly visible in each successful experiment. The topography of the two other maps was mirrored against that map. Electrophysiological recordings in the anterior and the posterolateral map revealed that all units responded to flashes and to moving bars. Mean directional preferences as well as latencies were different between neurons of the two maps. Tracing experiments confirmed previous reports on the thalamo-wulst connections and showed that the anterior and the posterolateral map receive projections from separate clusters within the thalamic nuclei. Maps are connected to each other by wulst intrinsic projections. Our experiments confirm that the avian visual wulst contains several separate retinotopic maps with both different physiological properties and different thalamo-wulst afferents. This confirms that the functional organization of the visual wulst is very similar to its mammalian equivalent, the visual cortex.

## Introduction

The visual system of birds, like that of other vertebrates, comprises two ascending visual projections, the tectofugal or collothalamic pathway and the thalamofugal or lemnothalamic pathway [1]. The tectofugal pathway is traditionally seen as the more important one, mainly because lesions of the thalamofugal pathway did not result in substantial effects on visual performance, while lesions of the entopallium, the telencephalic target of the tectofugal projection, affected basic visual functions like brightness and pattern discrimination [2,3] as well as complex tasks like category or "concept" learning [4,5].

The role of the visual wulst (VW), the telencephalic target of the thalamofugal pathway, has remained enigmatic for quite a long time. Early studies revealed no effects of lesions on pattern, color, or intensity discrimination, on visual acuity, and only small effects on reversal learning or repeated acquisition tasks. Electrophysiological recordings from owls [6] indicated that the visual wulst was involved in the integration of information from both eyes. Its functional properties were very similar to that of the visual cortex of mammals, comprising orientation selective binocular neurons, which were organised in a retinotopic manner.

In mammals, binocular neurons are providing depth information facilitating distance estimation and background-object differentiation [7]. Accordingly, Nieder and Wagner [8] found that binocular wulst neurons of barn owls are sensitive to horizontal disparity between the two eyes. In contrast to owls, however, only marginal binocular interaction has been demonstrated in laterally eyed birds such as pigeons, chicks, and zebra finches [9,10,11,12]. In addition, our own experiments indicate that binocular interactions in zebra finches are not comparable to those of owl binocular wulst neurons [13].

The discrepancies between the owl studies and those in pigeons and zebra finches are probably due to a different degree of binocular overlap. The owl eyes are located frontally with a high degree of binocular overlap, while the majority of other avian species comprise laterally placed eyes with only marginal overlap of the visual fields of both eyes [14]. It has been shown that the size of the avian visual wulst correlates with the amount of binocular overlap, and it has been speculated that this correlation may be due to the amount of binocular information processing within the visual wulst [15,16].

This, however, means that the visual wulst in birds with laterally placed eyes may be involved in other behavioral tasks rather than binocular processing. Indeed, it has been shown that the visual wulst is involved in spatial orientation [17], sun compass orientation [18], as well as in vision mediated earth magnetic field orientation [19,20]. In the present experiments, we tried to further elucidate the participation of the visual wulst in visual processing by investigating the functional organisation of this brain structure.

As mentioned above, the visual wulst is the telencephalic target of the thalamofugal pathway, which originates from the retina and projects to the nucleus geniculatus lateralis pars dorsalis (n. GLd) of the thalamus and further to the visual wulst (VW). The GLd is composed of various sub-nuclei of which the DLA (n. dorsolateralis anterior thalami), DLL (DLLd-dorsolateralis anterior thalami pars dorsalis and DLLv-dorsolateralis anterior thalami pars ventralis), LdOPT (n. lateralis dorsalis nuclei optici principalis thalami), and SpRt (n. suprarotundus) constitute the core portion, characterized by their input from the retina and projection to the VW. Visual wulst also receives input from another thalamic nucleus, the superficial parvocellular nucleus (SPC) which has not been shown as yet to receive retinal information (review: [21]).

As the name implies, the VW is characterized by its appearance as a bulge ("Wulst" is German for "bulge") in the forebrain, which caudally merges into hippocampus. In zebra finches, it is differentiated into three layers, the dorsal most hyperpallium apicale (HA), the intermediate nucleus interstitialis hyperpallii apicale (IHA) and the ventral most hyperpallium densocellulare (HD) [22]. It has to be mentioned here that we are using the names of the layers according to the new nomenclature by The Avian Brain Nomenclature Consortium [23], not the names used before the nomenclature change (e.g. [22,24]).

The three layers of the visual Wulst were differentiated by cell type anatomy. IHA is a granular layer and thus HA and HD can otherwise be referred to as supragranular and infragranular layers of the wulst (eg: [24]). The afferents from the thalamic nuclei terminate in the middle layer, the IHA, and the information is then transferred to the HA and the HD. HA serves as an output layer and passes the information to other ipsilateral telencephalic regions like intermediate arcopallium (AI) and lateral portion of the nidopallium frontale (NF). HA gives rise to the tractus septomesencephalicus (TSM), a descending fiber bundle carrying information to the thalamus and to the optic tectum. The HD comprises projections predominantly to the HA, dorso-caudal telencephalon and minor projections to other areas which also receive input from the HA [21,25].

Using optical imaging of intrinsic signals, we have previously shown that the visual wulst of the zebra finch, a laterally eyed songbird, contains at least two topographically ordered maps of the visual field [13,26,27]. We speculated that the existence of wulst multiple maps which are organised similar to the multiple representations in mammals might support the notion of homology between visual wulst and visual cortex. In all zebra finch imaging experiments, the most posterior map was visible, while the other wulst retinotopic maps, which were mirror images of the first one, could not always be found and were quite variable in both shape and position [13,26].

We have further shown that the posterior map does not span the entire visual field of the contralateral eye [13]. Instead, it represents only a small strip with an azimuth range of about 5° horizontally from the beak within the ipsilateral visual field towards about 150° from the beak into the contralateral field. The elevation of that field spans from about -5° below the horizon to about 25° above the horizon. This is different from the V1 representation in mammals, which comprises the entire visual field of the contralateral eye (e.g.: [28]. There was no evidence of a foveal magnification like it was shown in V1 of mammals possessing a fovea [29,30] although there is a deep fovea in the zebra finch retina [13]. However, we observed a small binocular overlap of ± 5° and also some interaction of inputs from the ipsi-and the contralateral eye (although the ipsilateral eye input was very small), indicating that some binocular processing is going on [13], but is probably not comparable to that shown in owls [6].

In the present experiments, we have collected a variety of additional data on the structure and function of the zebra finch thalamofugal system and especially the visual wulst which could probably help to further elucidate its functional role and to find out more about similarities and differences of the avian visual wulst and the mammalian visual cortex, respectively. As a first step, we have looked at the variability of the topographic maps, which is obviously bigger than that observed in comparable studies in mammals. However, it turns out that it is possible to find common properties of the different experimental cases, and we have identified at least three maps, which are localized relative to each other in a fixed pattern. We also conducted experiments with two fluorescent tracers injected into two of the three maps to investigate the topographic specificity of the thalamic afferents. Finally, we performed electrophysiological recordings from neurons of the posterior map and of the anterior map to get first information on the functional characteristics of the visual wulst neurons and about possible differences according to the position within the wulst. The present experiments support the view that the functional organisation of the visual wulst is very similar to that of the visual cortex in mammals, although its function is very dissimilar.

## Material and Methods

All experimental procedures were performed according to the German Law on the Protection of Animals and permitted by the local government. All imaging experiments, performed at the University of Göttingen, were approved by the „Niedersächsisches Landesamt für Verbraucherschutz und Lebensmittelsicherheit” (permission numbers 84–02.04.2011.A217 and 33.9-42502-04-14/1451), all other experiments, performed at the University of Bielefeld, by the "Landesamt für Natur-und Verbraucherschutz" (permission AZ 87–51.04.2010.A069 and AZ 9.93.2.10.36.07.105, LANUV, NRW).

### Optical Imaging

Procedures were performed as described previously [26,27]. Briefly, the birds were anesthetized by injection of 0.1 ml of 20% urethane intramuscularly. The birds’ heads were fixed using a stereotaxic head-holder for small birds [31], modified to hold the head in a position where the beak was oriented horizontally. A craniotomy was performed on the left hemisphere to expose the visual wulst, leaving the dura mater intact. The exposed brain area was then covered with warm agarose (2.5% in saline) and a glass cover slip. The contralateral eye was opened by retracting and fixating the lower eyelid in an open position by Histoacryl (Braun, Melsungen), keeping the nictitating membrane intact. If moistening by the nictitating membrane appeared to be not sufficient, silicon oil was applied to keep the sclera in good condition. At the end of the experiment, the birds were sacrificed by an overdose of sodium pentobarbital.

Neuronal activity in the left visual wulst of zebra finches was captured using autofluorescent flavoprotein imaging [27, 32,33]. To image sensory-driven activity, a temporally periodic stimulus was continuously presented to the bird and the brain’s response at the stimulus frequency was extracted by Fourier analysis [34]. Optical images of visual wulst activation were obtained using a CCD camera (Dalsa 1M30) and a 130 x 55 mm lens with an aperture of 1.2 (Nikon, Tokyo, Japan), controlled by custom software. The acquired image covered 4.6x4.6 mm of the brain surface. The camera was focused below the wulst surface at a depth of 500 μm, and neuronal activity was captured using blue light (455 ± 10 nm). Frames were acquired at a rate of 30 Hz, binned to 7.5 Hz and stored as 512x512 pixel images after spatial binning of the camera image.

A high refresh rate monitor (Flatron LCD 295LM, 100 Hz, 46.5×30 cm), covering 75°×50° of the visual field, was placed at an angle of 60° (position where the fovea is directed) at a distance of 30 cm from the eyes. Vertically, the monitor was positioned such that the horizontal line from the eye to the monitor was 7 cm above the lower rim of the monitor. Visual stimuli consisted of vertical bars moving horizontally back and forth (azimuth) or horizontal bars moving vertically (elevation), 4° wide and moving at a speed of 10°/s.

Activity maps were calculated from the acquired frames by Fourier analysis using custom software [34]. The phase component of the signal was used for the calculation of retinotopy and visualized in so-called phase maps, which are colour-coded with respect to the position of the stimulus eliciting this activation, with blue coding the center of the monitor. The amplitude component represents the intensity of neuronal activation, i.e. response magnitude expressed as fractional change in reflectance x 10^−4^. The activity distribution of each experiment is visualized in activity maps. The combined information of the magnitude of neuronal activation and retinotopy is displayed in so-called polar maps. Topographically ordered areas are characterized by the sequence of colours—blue which codes the center of the screen (the direction into which the fovea is "looking") flanked by red/violet coding the right and green/yellow coding the left side of the screen in the trials with azimuth stimuli. In the vertical, the horizontal plane (7 cm above the lower rim of the monitor) is coded by light/greenish blue and is flanked by blue-violet-red for areas dorsal and green-yellow for areas ventral from the horizontal. The colour codes used to display the retinotopic polar maps are shown along with each polar map.

As described in our previous paper [13], we also constructed iso-azimuth and iso-elevation line maps. Iso-azimuth and iso-elevation lines describe, similar to the colour codes used in the polar maps, the line of activation within the brain which is triggered by a vertical bar moving horizontally back and forth over the stimulus screen (azimuth stimulus), or the line of activation induced by a horizontal bar moving up and down (elevation stimulus). The numbers on the iso-lines indicate the corresponding viewing angles. Foveal direction (60°) is denoted as 0° for azimuth lines. For elevation lines, 0° denotes the direction of the horizon. Positive values are lateral from the foveal direction for the azimuth and dorsal for the elevation changes, negative values correspond to frontal direction for azimuth and caudal for elevation changes.

### Electrophysiology

Seventeen adult male zebra finches (*Taeniopygia guttata*) from the stock of the Bielefeld Behavioral Biology department were examined. Birds were anesthetized by an injection of urethane (SIGMA Diagnostics, 0.01 ml, 20% PBS) into the flight muscle. When reflexes on pinging the toes were not observed any longer, the bird’s head was attached to a stereotaxic head holder [31]. Xylocain Gel (2%, Astra Zeneca GmbH, Wedel, Germany) was applied to the skin of the ear holes for additional local anaesthesia.

Feathers were removed and the skin was incised and retracted to expose the skull at the desired positions for electrode placement. Then, the skull was opened by removing the two bone layers. The dura was incised using a sharp injection needle at the coordinate determined for electrode insertion. The eyelid of the right eye was fixed by surgical adhesive in an open position shortly before the experiment started. The nictitating membrane was kept intact to protect the eye from desiccation during the experiment. During anaesthesia the nictitating membrane is open and blinks in response to slight touch stimulation.

The reference electrode was clamped to the skin of the head and moistened with saline (0.9%). The recording electrode (tungsten in glass micro-electrode TM31A10, World Precision Instruments, Inc., Sarasota, USA, 0.9–1.0 MΩ, tip diameter 1–2 μm, 1 μm insulation) was positioned according to the coordinates of the topographic maps which were determined in previous imaging studies [13,26] using the coordinate system of the stereotaxic atlas of the zebra finch [35]. After penetration of the dura, a grounded hood of fine wire mesh was attached to the stand to shield bird and electrode from electromagnetic noise caused by the stimulus screen. The hood did not essentially obscure the bird’s view.

Visual stimuli were provided by a computer screen with 100 Hz refresh rate located at the same position relative to the experimental animal as the stimulus screen in the optical imaging experiments (see above). Stimuli identically to those used in the optical imaging experiments were produced with help of the Vision Egg library [36]. White stripes of 5 cm width were moving into four directions (up, down, left, right) over the screen with a speed of 4.1 degrees/ second (see optical imaging methods). In addition, we were using short flashing illumination of the whole screen during the searching phase when advancing the electrode, and also for determination of the latency of isolated neurons. 40 sweeps were performed for each direction and latency determination.

The electrode was advanced slowly in steps of 2 μm by a motorized micro drive. The amplified neuronal responses were visualized by an oscilloscope and also monitored acoustically by a loudspeaker. When a neuron was responding to the moving flashlight, its responses were tested as described below. After reaching a depth of 2000 μm, the electrode was retracted completely and re-inserted at a slightly different coordinate for a new approach. The distance between recording sites was at least 50 μm to avoid recording the same neuron twice.

The received signal was amplified (x1000) and band pass filtered (300 Hz lower, 20 kHz upper cut-off frequency; A-M Systems Model 1800) before it was digitized (CED 1401mkII, Cambridge Electronic Design) and stored ("Spike 2" recording software, Cambridge Electronic Design).

The activity of single neurons within a recording was separated offline using the spike sorting function provided by Spike 2 (a template matching procedure). The resulting files were further analysed with custom made Matlab (Mathworks) scripts including a separately published toolbox ‘CircStat’ [37].

At the end of each experiment, the birds were sacrificed by an overdose of sodium pentobarbital and the position of the last electrode track was marked by an electrical lesion. The brain was removed and stored for at least two days in fixative (4% paraformaldehyde in phosphate buffered saline (PFA) followed by 30% saccharose in PFA). Coronal 40 μm sections were cut, mounted on glass slides and stained with Giemsa dye (SIGMA Diagnostics, St. Louis, USA).

### Neuroanatomy

13 male zebra finches from the institute’s stock were used for this study. The birds were anaesthetized by 0.04 ml of equithesin [38] and placed in a stereotaxic device especially constructed for small birds [31], which secures the head in the same position as depicted by the stereotaxic atlas of the zebra finch [35]. After removing the feathers, making a cross like incision to the skin and pulling the skin flaps aside, a section of bone and dura was removed from the skull to expose the visual wulst of the bird at the left side of the brain or on both hemispheres depending on the intended experiment. Injections of fluorescent tracers were made using glass micropipettes, with tip diameters of 20–30 μm, which were advanced into the brain using a Narishige microdrive. Injections were then performed with a custom made pressure system comparable to the commercially avilable "picospritzer". Two different fluorescent dextrans were used, either micro-ruby (red) or micro-emerald (green) (both 3,000K molecular weight; Molecular Probes, Eugene, OR). We attempted to inject one of the tracers in the posterior retinotopic map which was visible at the same place quite reliably in any of our experiments; the second injection was aimed towards a more anterior map which was also visible in many, but not in all experiments, and the location of which was somewhat more variable. As already described for the electrophysiological recordings, the micropipettes were positioned according to the coordinates of the topographic maps which were determined in previous imaging studies [13,26] using the coordinate system of the stereotaxic atlas of the zebra finch [35]. We also made experiments with coordinates outside of the anterior or the posterior map, also one experiment where we injected into the right instead of the left hemisphere, two bilateral injections at coordinates between the anterior and the posterior map, and two retinal injections. However, the results of these injections will not be reported here in detail because there were too few cases to draw reliable conclusions.

In four cases, optical imaging was done as already described above, prior to the tracer injections. The only exception was that we used halothane for anesthesia because the birds had to wake up at the end of the experiment for a survival period of 24 hours. We then determined the location of the maps by overlaying the brain surface vascular pattern (taken by the CCD camera device of the optical imaging setup, using a green filter which intensifies the contrast) and the retinotopic phase map. Using the overlaid vascular pattern and the phase map image, we then propagated the glass pipette with a stereotaxic microdrive to the desired positions, advanced the tip into the brain by 500 μm (the depth of the input layer of the visual wulst) and injected the tracer with help of a nanoliter injection device (General Valve, Fairfield, NJ).

After the injections were made, the craniotomy was covered with the skin flaps which were then agglutinated using medical cyanoacrylic glue (Histoacryl), and the wound was anaesthetized by local anaesthesia (2% xylocaine gel). 24 hours later the birds were deeply anesthetized with sodium pentobarbital (100 mg/kg) and immediately perfused transcardially with phosphate-buffered saline (PBS; 0.9% NaCl, 0.1 M phosphate buffer) followed by 4% paraformaldehyde in 0.1 M PBS (pH 7.4). The brain was removed from the skull and immersed in paraformaldehyde for another 24 hours at 4°C. For cryoprotection, the brain was then placed in 30% sucrose in 0.1M PBS until it sank to the bottom of the jar.

Using a freezing stage microtome, 40 μm coronal sections were cut and stored in individual wells containing PBS. The sections were mounted on gelatinized glass slides, dried, coverslipped using SlowFade Gold antifade reagent with DAPI (Invitrogen, Eugene, OR).

Sections were viewed with a compound light microscope (Zeiss Axioscope 2plus) equipped with the appropriate fluorescence filters (rhodamine and FITC). Images (5184*3456 pixels) were acquired using a Nikon digital camera under remote control by an IMac computer. Brightness and contrast of the photos were adjusted in some cases by Adobe Photoshop. Overlays of the fluorescein and the rhodamine pictures were made by using the "merge channels" function of the NIH "ImageJ" program.

## Results

### Visualizing wulst retinotopic maps by intrinsic signal optical imaging

Basic features of our optical imaging experiments were already described in previous papers [13,26,27]. Visual stimulation with moving bars resulted in activation of distinct areas of the visual wulst, which turned out to be topographically ordered neuronal maps, visible best in a depth of 500 μm, and outspread parallel to the brain surface. It was not possible to relate the maps to a distinct layer; but because the map quality became worse when we focussed the camera deeper (1000 μm) or more superficial (100 μm) we presume that the input layer IHA may be the source of the mapped activity, but that the topographical order extends to HA and HD also (see also [13]). In all successful experiments, we obtained at least one retinotopic map, here referred to as the posterolateral map, because additional maps not always obtained were located more anterior and medial to this map. In 17 out of 32 experiments, imaging revealed a single map, 13 experiments visualized multiple maps, and 2 just a faint second map in addition to the posterolateral one. In addition to these experiments with wild type birds, we also recorded topographic wulst maps in 6 individuals of the white morph of the zebra finch which has been shown to express a slightly deviating visual system [39,40]. In 5 of the 6 birds, there were multiple wulst retinotopic maps, while the 6^th^ bird had only one map. Because the other results concerning the white birds were not different from those of the wild type (coloured) animals, we do not present them separately.

**Fig 1:**
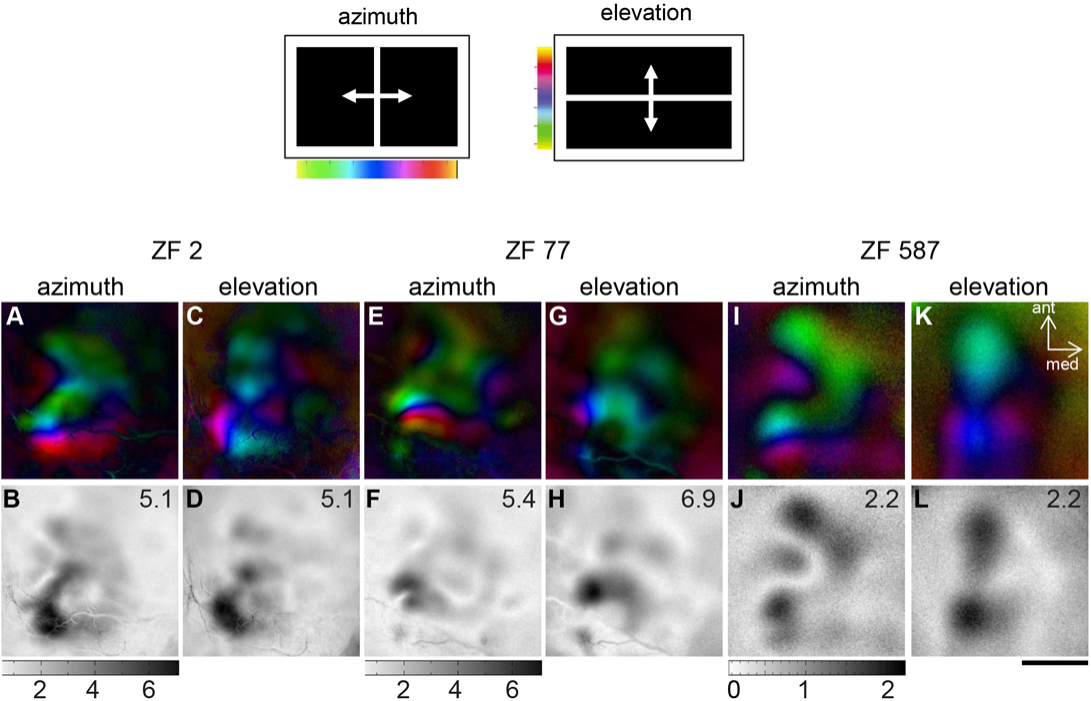
Optically imaged retinotopic maps in the zebra finch visual wulst are variable. Activation in the left visual wulst was induced by visual stimulation of the right eye with moving vertical and horizontal bars (top). Colour coded polar maps of azimuth and elevation (upper row) and grey-scale coded activity maps (lower row) from three zebra finches (ZF 2, ZF 77 and ZF 587), showing at least three separate representations of the visual field. The magnitude of stimulus-driven neuronal activation is given as a number in the upper right corner of the activity maps. **A**/**B**, **E**/ **F**, **I**/ **J:** azimuth and activity maps from three different birds, showing interindividual differences in both retinotopic details and activation strength. **C**/**D**, **G**/ **H**, **K**/ **L:** elevation and activity maps from the same birds as above. Note that e.g. the anterior retinotopic maps in **C** are mirror images of the posterior retinotopic map. Scale bar 500 μm.

Besides this variation in numbers, there was quite a big variation in the exact appearance of the single maps and in the relation of the maps to each other. However, comparing all experiments, we were able to define an arrangement of three maps, which was found in 9 of the 13 experiments emanating multiple maps. Fig 1 provides examples of multiple maps from three different zebra finches. The upper panel of Fig 1 shows colour-coded polar maps of retinotopy (colour-code shown on top of the figure; for further description, see methods), displaying retinotopic organization as a sequence of colour for both azimuth and elevation; the corresponding activity maps are illustrated in the lower panel. Both azimuth and elevation maps clearly show at least three distinct representations of the visual field of the contralateral eye. It is obvious that the distribution of the maps within the imaged area was not identical from brain to brain; however, in all cases, a topographical mapping was visible in the lower left corner corresponding to the posterolateral part of the visual wulst. This posterolateral map was the most stable one, it was detected in every successful imaging experiment. Another more variable map was detected anterior to the posterolateral map. Because it was in most cases slightly displaced medially, we call it the anteromedial map. A third, even more variable map, was identified medial to the posterolateral map (posteromedial map). The lower panel of Fig 1 depicts the activity distribution within the recorded wulst area. It shows that the posterolateral map area was always strongly activated by the visual stimuli, while the activation of the other maps was quite variable, as was the position of the activation maxima.

**Fig 2:**
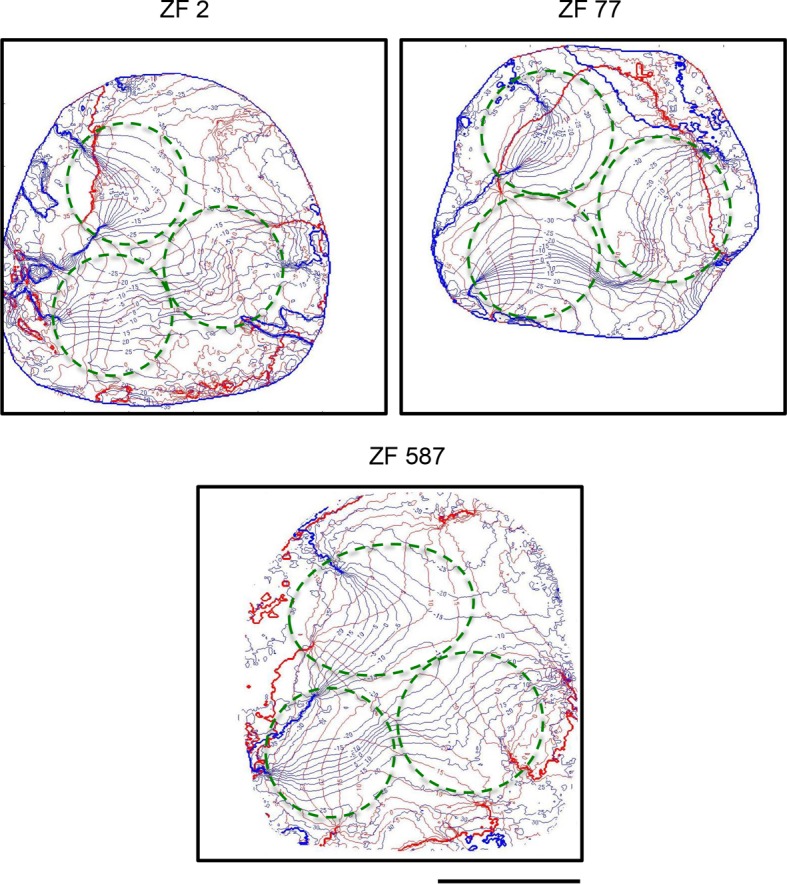
Iso-azimuth and iso-elevation line maps from three examples of multiple maps in the visual wulst. Iso-azimuth (blue) and iso-elevation lines (red) from the same birds shown in Fig 1 are illustrated. The roughly parallel arrangement of iso-azimuth and iso-elevation lines indicates an orderly retinotopic representation of the multiple maps, indicated by green circles. Note that iso-azimuth and iso-elevation lines tend to be perpendicular to each other. Scale bar 500 μm.

Fig 2 shows the iso-azimuth and iso-elevations lines of three examples of multiple retinotopic maps, marked by green circles. Each iso-line on the maps indicates the longitude (azimuth maps) and latitude (elevation maps) of the visual field area, which evoked activity in the visual wulst. The maps centers are characterized by iso-azimuth lines that are quite parallel to each other. The iso-elevation lines, which are also parallel to each other and roughly perpendicular to the iso-azimuth lines, are more widely spaced in all three maps, a feature which we have described already for the posterolateral map in a previous paper [13].

Another common feature was the different orientation of the three maps (Figs 1 and 2). In the posterolateral map, the gradient along the fronto-lateral axis of the visual field was oriented rostro-caudally, while the dorso-ventral axis was oriented latero-medially. The posteromedial map was rotated anticlockwise against the posterolateral map by ~ 45°, and the topographic relation between the frontolateral and the dorsoventral axes was preserved. Compared to the posterolateral map, the azimuth representation within the anterior map was rotated clockwise by ~ 90°, and so were the iso-elevation lines, which were also mirrored compared with those of the posterolateral map. Although we did not make measurements, it might be useful to point out that the spacing of the elevation lines of the posteromedial map appeared to be similar to that of the posterolateral map, in which the elevation spacing was more than double of the azimuth line spacing [13]. Likewise, the spacing of the iso-elevation lines appeared to be even wider in the anterior map.

**Fig 3:**
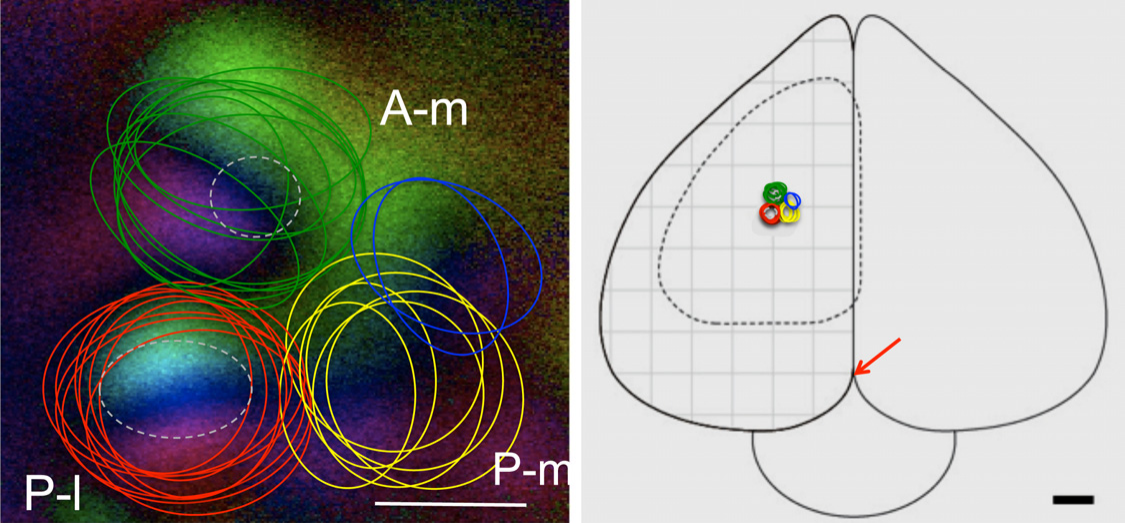
Visualization of the interindividual differences in multiple map locations. Left panel: Superimposition of the retinotopic map positions from different experiments. Coloured circles circumvent the centers of all imaged wulst retinotopic maps in 9 zebra finches: the posterolateral map (P-l, red), the antero-medial (A-m, green) and the postero-medial map (P-m, yellow). In some cases, a fourth map was discernible (blue ovals). White dotted circles indicate the location of the electrophysiological recordings and the tracer injections. Scale bar 500 μm. Right panel: Top view of the zebra finch brain illustrating the location of the maps. Arrow: origin of the coordinate system used. The grid has a width of 1mm. Scale bar 1mm [35].

Fig 3 illustrates the interindividual variability of the map positions obtained in the different imaging experiments. We marked the approximate positions of the retinotopic maps by coloured circles in the azimuth polar maps (these were chosen because they best show the spatial limits of the maps) obtained in the experiments and then superimposed the coloured circles onto one exemplary multiple wulst map. It is obvious that the position of the posterolateral map (red circles) is the most stable one, those of the anteromedial (green circles) and the posteromedial (yellow circles) are more variable. Blue circles depict the position of a fourth map, which we saw in only a few experiments. The hatched circles in Fig 3 depict the areas that we subsequently investigated by extracellular electrophysiology (see below). The properties of the other two areas were not further investigated in this series of experiments.

### Electrophysiology

**Fig 4:**
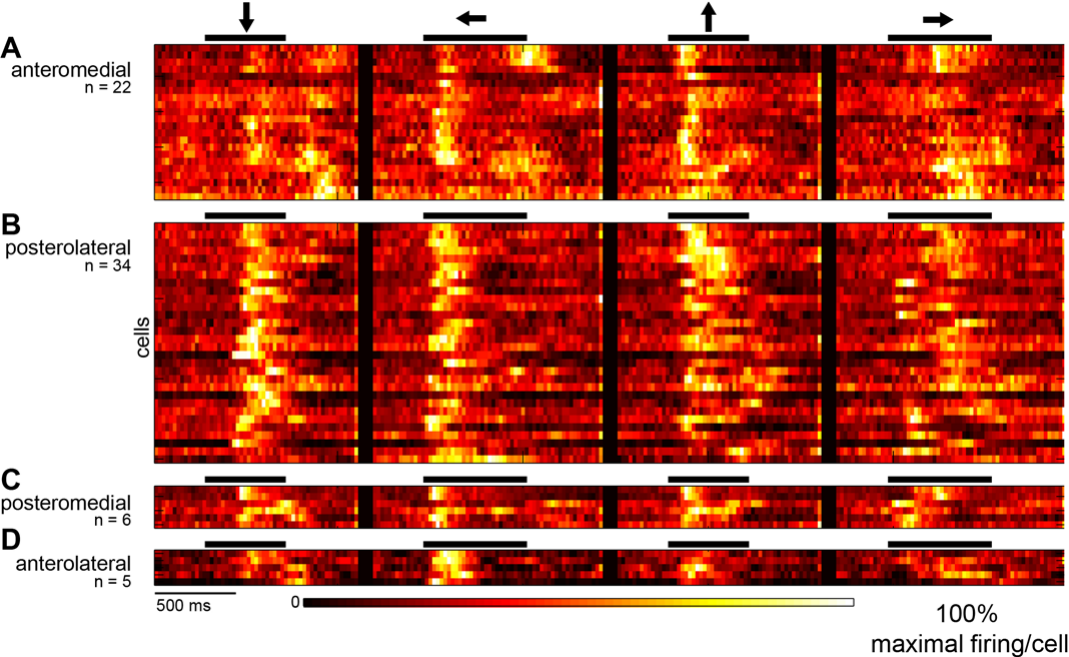
Visual response properties of neurons in different visual wulst regions: Overview of the electrophysiological recordings. The neuronal responses of each recorded cell to each visual stimulus is displayed after averaging over 40 trials and normalizing to the maximum firing rate of the cell. Rows correspond to cells, time is on the x-axis. Dark colors correspond to low firing rate, hot colors to high firing rates. Each block (A-D) comprises cells from the four different wulst regions in which we sampled (C and D are not further treated in the paper because the samples were too small). Black bars above each block indicate the stimulus duration (the transition of the moving bar across the monitor, 0.48 sec for vertical motion, 0.64 sec for horizontal motion), and arrows show the direction of the bar movement.

Fig 4 shows an overview of the electrophysiological recordings. Overall, 74 of 88 neurons responded reliably to flashes as well as to moving bars (see methods). The response to the four stimulus directions is shown for each recorded neuron, averaged over 40 trials and normalized to the maximum firing rate. Rows correspond to neurons; time is on the x-axis. Dark colors correspond to low firing rate, hot colors to high firing rates. Each block shows cells from the four spatial regions in which we sampled, indicated to the left. Black bars above each block indicate the stimulus duration (the transition of the moving bar across the monitor, 0.48 s for vertical motion, 0.64 s for horizontal motion), and arrows show the direction of the bar movement. It is obvious that the responses of neurons are not uniform: while all neurons exhibited a response starting at the onset of the stimulus, only 27 also exhibited an off-response. Onset and offset is not identical with the leading or trailing edge of the stimulus. Instead, the neurons were activated when the moving bar entered the receptive field of the recorded neuron, and the off response was, with some delay, elicited when the bar was leaving the receptive field.

**Fig 5:**
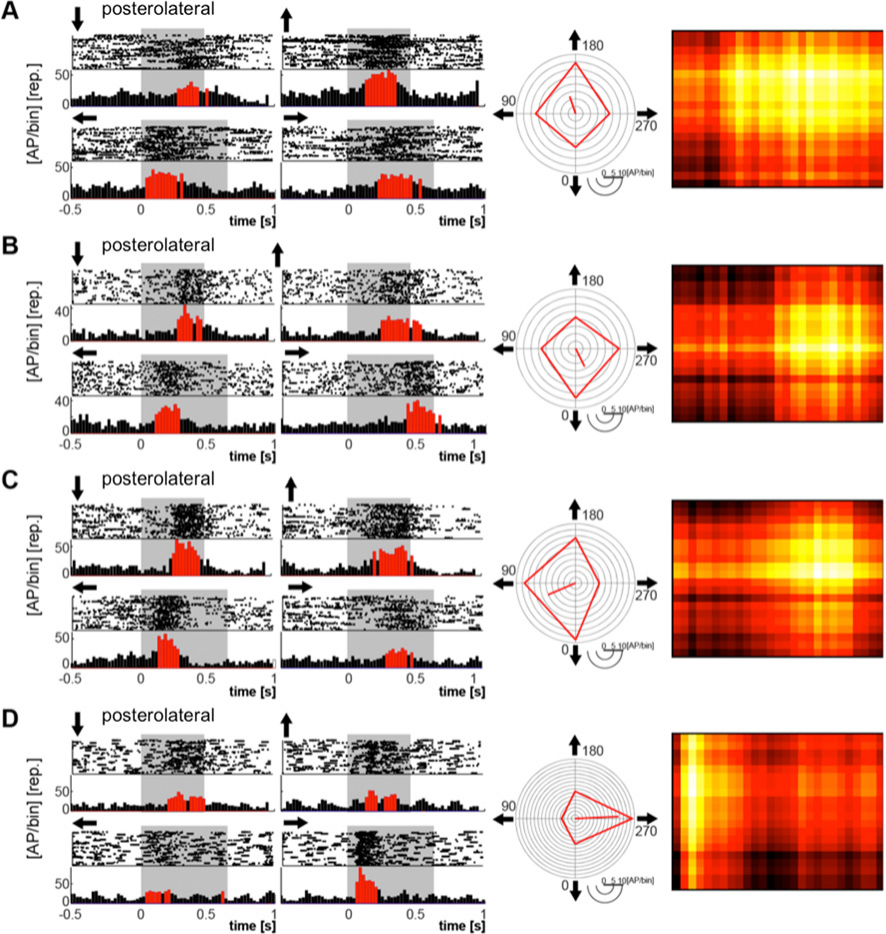
Four examples of neurons from the posterolateral wulst area with on–responses (A-D). Left two panels: Raster plots (40 sweeps) and PSTH´s. Grey shaded areas: Time of stimulus transition across the monitor. Arrows indicate the direction of stimulus movement. Medial panel: Directional responses of the same neurons as circular diagrams. The line originating in the center is the main vector. Its length is a measure for the directionality of the neuron. Right panel: Estimation of the receptive fields by combining the responses to all four directions. Light colors indicate strong activation.

**Fig 6:**
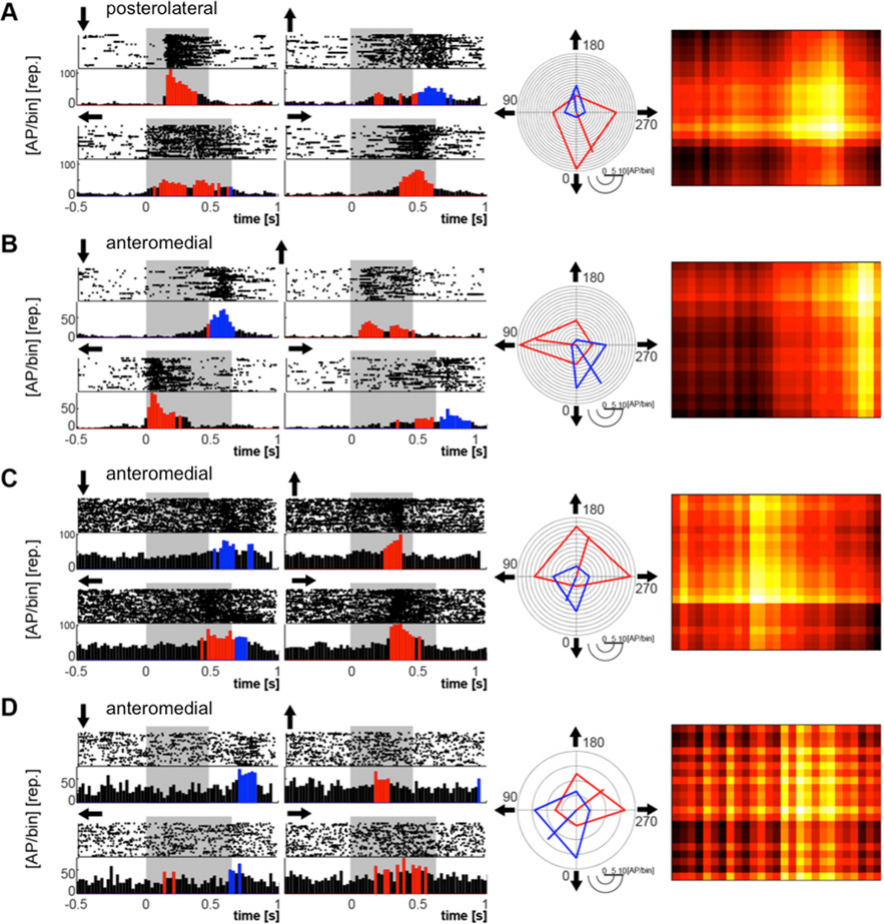
Neurons from the posterolateral and anteromedial wulst areas with on (red) and off (blue) responses. Note that the off responses are often maximal with a stimulus direction opposite to that of the on–response. Other features as in Fig 5.

Examples of both types of neurons are depicted in Figs 5 and 6. In both figures, the left panels show raster plots and peristimulus-time histograms (PSTH) of four examples each with on-responses (Fig 5) and with on-off responses (Fig 6). The shaded area depicts the time of visibility of the stimulus on the stimulation monitor. Red bars in the PSTH indicate an activation two standard deviations above background. The highest amplitudes within these activation bouts were used to construct the circular diagrams in the medial panels of Figs 5 and 6, illustrating the differences in direction preferences between the different neurons. Red lines connect the maximal "on" responses to each of the four stimulus directions, the blue lines in Fig 6 those of the "off" responses. It is obvious that most neurons were, to different degrees, direction selective. The line emanating from the center is the mean vector calculated from the four directional responses. The right panels in Figs 5 and 6 show a rough reconstruction of the receptive field of the given neuron, which we made by combining the "on" responses to all movement directions. The size of the receptive fields differed from quite small ones (eg. Fig 5D) to very big ones that were extending over the limits of the stimulus monitor.

**Fig 7:**
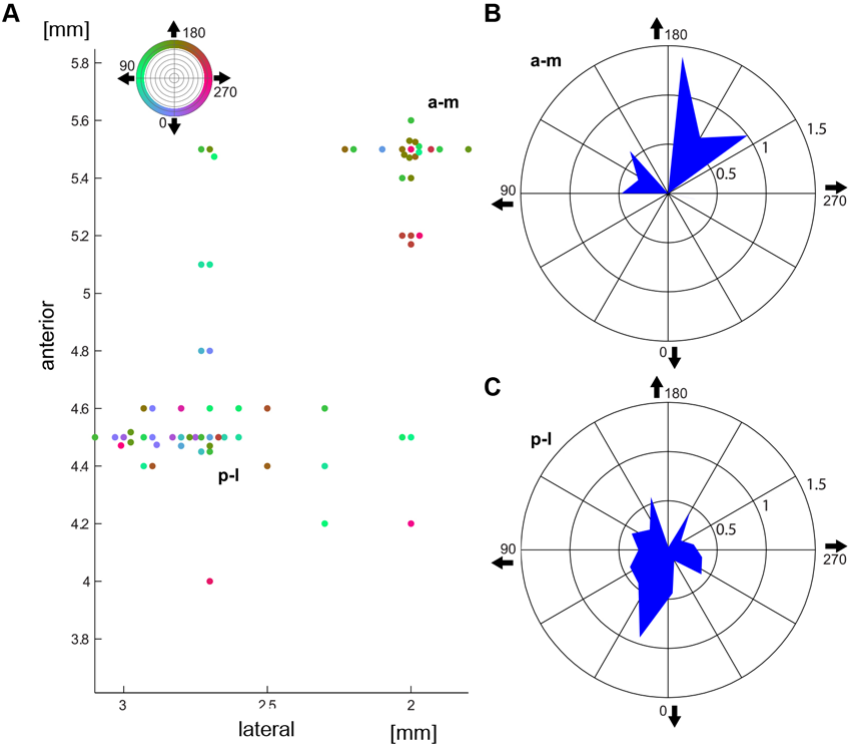
Distribution of directional responses within the anteromedial (a-m) and the posterolateral (p-l) wulst field. A: Positions of the recording sites on the surface of the visual wulst of the left hemisphere. x-axis depicts the distance from the midline of the brain, y-axis the distance from the Y-point (see methods). The compass rose at the top left indicates the colour coding of the different directions used to mark the directions at the recording sites. B: directional response distribution of the anteromedial map, 0 degrees: downwards, 180 degrees: upwards, 90 degrees: forwards (rostral), 270 degrees: backwards (caudal). C: posteromedial map, explanations see B. Red colour indicates on responses, blue colour indicates off responses.

Fig 7A shows the spatial distribution of our recording sites on the visual wulst as seen from above. Most recordings were located within the posterolateral and the anteromedial area (p-l and a-m, see also Fig 3). The colours of the recording points code the direction of the mean vector (coloured circle, the direction of movement of the stimulus bar on the computer screen is indicated by the arrows). The distribution of the coloured recording points already indicates that the direction preferences differed between the two areas. This is quantified in the two circular diagrams on the right (Fig 7B and 7C), which show the distribution of the directionality vectors calculated from the direction preferences of the single neurons. While the antero-medial map (Fig 7B) shows maxima with upward directions, the maximum in the postero-lateral map (Fig 7C) is directed downwards. This also indicates that the preferred orientation of the bars in both cases may be horizontal; however, it is not possible to really disentangle the orientational and directional preferences on the basis of our experiments because we did not measure both parameters independently.

**Fig 8:**
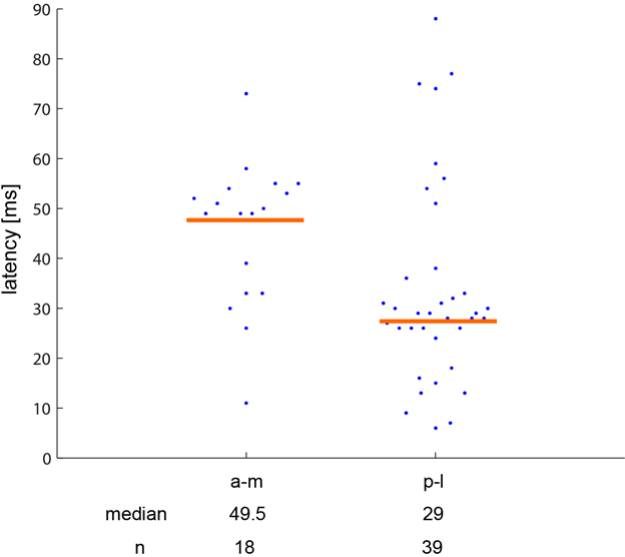
Latencies of neurons within the anteromedial (a-m) and the posterolateral (p-m) map. Blue dots: individual latencies, horizontal orange line: median.

In addition to directionality, the two areas also differ in the latency of neuronal responses as measured by flashing illumination of the entire stimulus screen (Fig 8). The neurons of the anteromedial area comprise significantly (Mann Whitney U-test, 2-sided, z = 2.07, p = 0.0385) longer latencies (median 49,5 ms) compared with those of the posterolateral ones (median 29 ms).

### Tracing

Our tracing attempts were not aimed to provide a complete analysis of the projection patterns of the thalamic visual nuclei to the visual wulst. Instead, we focused on investigating whether the posterolateral and anteromedial maps received distinct afferents from thalamic regions. We restricted our analysis to those maps (in the left hemisphere) because they were also investigated with electrophysiological methods (see above).

Principally, our results are congruent with those previously described by other authors (e.g. [22,41,42,43]) and reviewed by Güntürkün et al. [21]. We here found projections to the visual wulst from the following thalamic areas: SPC (n. superficialis parvocellularis), DLAmc (n. dorsolateralis anterior, pars magnocellularis), DLLd and DLLv (n. dorsolateralis pars dorsalis/ pars ventralis), LdOPT (n. lateralis dorsalis nuclei optici principalis thalami), n. SpRt (n. suprarotundus).

Most of these nuclei projected to the ipsi- and the contralateral visual wulst, as has already been shown in the above mentioned papers. In general, the strength of the projections to the anteromedial map was smaller than of those to the posterolateral one, but there was no case where we could find a projection exclusively to the one or the other area. On the other hand, there were no single neurons projecting to both wulst areas, as we did not find any colocalization of the two tracers within individual neurons of the thalamic nuclei.

**Fig 9:**
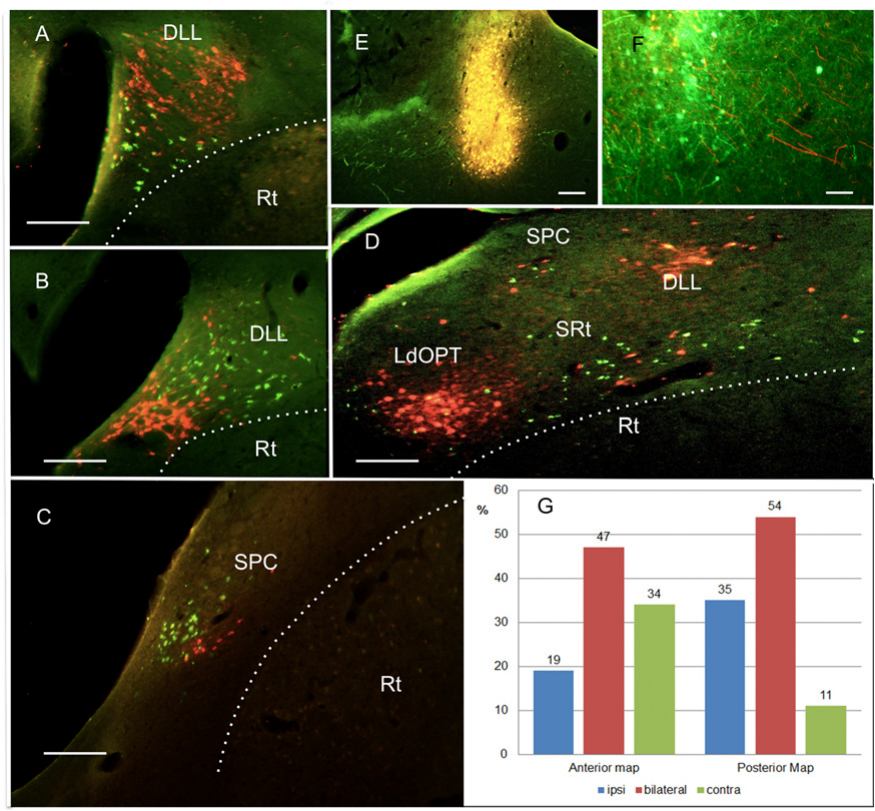
Tracing of thalamo-wulst afferents into the posterior and anterior map. **A-D**: Retrogradely labeled neurons within various thalamic relay nuclei of the thalamofugal system. Scale bars, 50 μm. **E, F**: Wulst injection sites with efferents from the second injection site in the other retinotopic wulst map. Scale bars 100 μm. For details see text. **G:** Percentage of ipsi- (blue), contra- (green) and bilateral (red) projections to the anterior and the posterior wulst map.

Within the thalamic nuclei, there were clusters of neurons projecting exclusively to one of the two areas (Fig 9A–9D), indicating that these nuclei comprise separate subdivisions serving only one of the two wulst maps. There was no obvious pattern of systematic arrangement like medial to lateral or anterior to posterior. The clusters were found in both hemispheres, confirming earlier studies that the thalamo-wulst projection is bilateral. Fig 9A shows an example (Zf45) where the dorsal part of DLL contains red fluorescent neurons retrogradely labeled from the anterior map (rhodamine injection) while the ventral part shows green fluorescent neurons labeled from the posterior map. Fig 9B shows the same area in another case (Zf 56) where we injected the green tracer into the anterior and the red tracer into the posterior map, and got a very similar picture just with interchanged colours. Fig 9C depicts a clustered label within SPC where the neurons projecting to the anterior map are labeled in red, and those projecting to the posterior map in green (Zf 320). In areas SRt and SPC, we also observed non-clustered, overlapping projections: Fig 9D shows a case (Zf 45) where neurons retrogradely labeled from the anterior or the posterior map are intermingled. As mentioned before, there was no case of double labeled neurons, which would have been expressed here in yellow.

Our tracing experiments also revealed that there are interhyperpallial connections between the two visual representations. At both injection sites, anterogradely labeled fibers from the other injection site were visible (Fig 9E and 9F), indicating that connections between these two areas are reciprocal.

To get some quantitative information on possible differences between the afferents of the two maps from the ipsi- and contralateral side, we selected all cases with successful injections into the two areas, and counted how often we observed projections exclusively to the ipsilateral or to the contralateral hemisphere, or bilateral projections. Fig 9G illustrates the percentages of ipsi-, bi- and contralateral thalamic efferents to the anterior and the posterior map, respectively. Obviously, while almost half of the map afferents were bilateral (around 50%), the anterior map received more contra- than ipsilateral projections, and the posterior map more ipsi- than contralateral ones. This difference was significant (Chi squared test on the original numbers, df = 2, Chi squared = 8.77, p<0.05).

## Discussion

In the present and previous papers, it has been shown that the architecture of the visual wulst of laterally eyed birds is very similar, but not identical with that of the mammalian visual cortex. It has a layered structure, but this has evolved in a different way than the cortical layers in mammals [44,45]. The visual part of the wulst comprises several visual field representations like the mammalian cortex, but the intrinsic organisation of the maps is different ([13], this paper): Examples are i) the lack of a foveal magnification despite of the existence of a retinal fovea with higher ganglion cell density, ii) the extent of the visual field representation, and iii) the difference between azimuth and elevation magnification factors. The distinct visual field representations receive input from distinct subdivisions of the visual thalamic relay nuclei of both hemispheres. This feature is reminiscent of the thalamocortical connections of the mammalian visual system, but a direct comparison is hardly possible, mainly because there are not enough data. Visual wulst neurons are orientation and direction selective, but there is, as far as we know, no organization like ocular dominance or orientation stripes or pinwheels as observed in mammals [46,47] and in more frontally eyed birds like owls [6,48]. Although the equivalence of the mammalian geniculocortical and the avian thalamofugal visual system therefore remains a matter of debate, our present findings on the functional organization of the zebra finch visual wulst support the idea of a high similarity of this structure with the mammalian visual cortex.

We shall discuss here the main findings of our experiments in the light of the relevant avian literature and will go on with a more detailed comparison of features of the avian visual wulst with the mammalian visual cortex. To do this, we will predominantly cite articles from the seventies of the last century, because they dealt with very basic visual features, which we have been investigating here.

The present paper affirms that the visual wulst of the zebra finch, a laterally eyed bird, contains multiple topographic representations of the visual field. It is impossible that the arrangement of maps which we have obtained by optical imaging is due to some folding of the map as it has been proposed by Wilson [10]: The same part of the visual field (in our experiments the area which is focused by the fovea and its surrounding) is represented several times within the visual wulst, and changing the depth of our imaging plane resulted in a change in the quality of the map (the maps were in any case optimal at depth between 500 and 1000 μm), but not in a different representation pattern. The presence or absence of the anterior and the posteromedial map was not dependent on the depth of the imaging focus; we presume that these two maps were not optimally activated by our stimuli and therefore needed better physiological conditions of the experimental bird to be expressed properly (see also [49] for a discussion of this issue on the basis of imaging studies in mice). Our electrophysiological experiments also provide evidence that the response properties of the neurons within the posterolateral and the anteromedial wulst areas are different.

Our experiments show that iso-elevation and iso-azimuth lines are nicely ordered within the centers of the maps and also oriented perpendicular to each other. In all of the maps, the elevation iso-lines were spaced more widely than the iso-azimuth lines, which indicates a higher spatial resolution of the vertical. The same phenomenon has been observed in the mouse visual cortex using optical imaging of intrinsic signals [49]. While different azimuthal and elevational spacing has also been described in the mammalian literature (review: [50]), we have—as yet—no conclusive explanation why the vertical aspects of a visual object should be differently represented from the horizontal ones. There is a possible relation of our finding with that of Ng et al. [51] who showed in the pigeon that the visual wulst comprises an overrepresention of vertical orientation preferring neurons and tried to explain it with an adaptation to the properties of the visual flow field during flight.

We did not detect an overrepresentation of the fovea in any of the examined wulst maps although the zebra finch retina comprises a well developed fovea [13]. This could be due to the fact that we here stimulated only a limited visual field area around the fovea, but we have shown in our previous paper [13] that there is indeed no foveal overrepresentation at least in the posterolateral map. This is in contrast to findings in mammals where the fovea is overrepresented in area 17 (V1) of all foveate animals investigated so far, and it has been speculated that such overrepresentation correlates with the density of the retinal receptors [52,53]. However, quite a lot of higher order areas within the visual cortex do not comprise a foveal overrepresentation even if it is visible in V1 (review: [50]). We have speculated that this lack of foveal overrepresentation indicates that object identification is not the predominant task of the zebra finch visual wulst [13].

The exact location of the borders between the maps could not be examined in our experiments, as we were not able to present visual stimuli from the entire visual field with our setup, and thus shall not be discussed here. If one looks, however, at the centers of the map, it is obvious that the anterior and the medial posterior map are mirror images of the posterolateral map, a feature that can also be found in the mammalian visual cortex [49,54].

Our electrophysiological recordings demonstrate that most of the visual wulst neurons were direction sensitive to different degrees (Fig 5), and in neurons with on—off responses, the off response often was strongest to a direction opposite to that of the strongest on-response (Fig 6). Receptive fields, as far as we can judge from our gross estimations, were very variable; we did not find a sign of changes of receptive field size with depth. Thus, in contrast to owls, where Pettigrew and Konishi [6] demonstrated the existence of some ordered system of the distribution of binocular input and Liu and Pettigrew [48] found a pinwheel-like organization of orientation selectivity (as it had been shown before in the cat [55]), we were not able to detect a comparably ordered system in our experiments. This is in agreement with Wilson [56] and Ng et al. [51] who also found that the majority of the wulst neurons was orientation selective, but also did not detect organizational rules similar to those in mammals in their studies of the chick and the pigeon wulst. However, a lack of such organization has also been shown in some mammals [57]. In any case, not finding an orderly pattern does not mean that there is no organization at all; it may be present but not as easy detectable as those mentioned above.

Our results clearly show (Fig 7) that there is a difference between the posterior and the anterior map concerning directionality, but the neurons of both maps comprise the same (± horizontal) orientation preference. The latter is somewhat inconsistent with findings of Ng et al. [51] in the pigeon who claimed that vertical orientation preference was predominant among the wulst neurons. As stated above, however, we did not uncouple orientation and direction preferences in our experiments, so it is possible that we had got similar results as Ng et al. if we had, in addition to our stimuli, also presented vertically oriented bars with vertical movement and horizontal ones with horizontal movement. In mammals, it has been shown that there is an overrepresentation of horizontal as well as vertical orientation preferences (e.g. [58,59]). These authors explained this observation by a dominance of horizontal and vertical features within the visual environment. Ng et al. [51] presumed that the asymmetries in orientation or direction preferences may be due to asymmetries in the occurrence of horizontal or vertical optic flow during flight.

Quite unexpected were the latency differences between the neurons of the two maps, with that of the anterior map being almost double of that of the posterior map. Bredenkötter and Bischof [12] described visual wulst evoked potential peaks at around 30 and 60 ms, close to the values, which we obtained here with multiunit recordings, and showed that this difference is not an artifact. Latencies of the pigeons’ wulst neurons were reported to be in the range of 18–20 ms [11,60,61]; the difference between those measurements and our first peak (29 ms) may be explained because we used the peak latencies, while the other authors may have reported the "first deflection", the first visible increase of the spike frequency after presentation of a stimulus. This, however, cannot be the explanation for the very much delayed (49.5 ms, which roughly coincides with a second peak obtained in the evoked potential study of Bredenkötter and Bischof [12]) latency of the anterior map neurons. The difference also cannot be explained by afferents of differing length or more synapses between photoreceptors and the target area, as the tracing experiments show that the thalamic afferents stem from different subdivisions of the same thalamic nuclei. It can most probably also not be due to a secondary excitement of the neurons of the anterior area triggered by activation of the neurons of the posterior area via the intra-wulst connections, because the distances between the areas are too short to lead to such latency differences. We are therefore at present speculating about a feedback connection leading from the posterior map back to the thalamus and there triggering the excitation of neurons sending axons to the anterior area. This is as yet pure speculation, but with a combination of retrograde and anterograde tracing, it might be possible to verify or reject this idea.

According to our tracing study, the afferents of the two topographic maps are very similar in that both areas are receiving projections from the ipsi- and the contralateral thalamus, and we did not find any of the thalamic nuclei projecting to only one of the two areas. However, it was evident that within the thalamic nuclei, there were subdivisions projecting exclusively to one or the other map, and also some subdivisions projecting to both. It is quite difficult to compare the thalamic situation in birds with that of mammals. While the avian thalamic relay consists of several subnuclei as described in the introduction (although in owls these nuclei are much more clustered than in laterally eyed birds), the predominant mammalian thalamic relay is a laminated nucleus, the LGN (lateral geniculate nucleus) which includes the medial interlaminar nucleus (MIN). Additional afferents of the visual cortex stem from the so called geniculate wing of the pulvinar-lateralis posterior complex [62]. Despite the differences, the pattern we have shown here is reminiscent of that seen in mammals. In the cat, the LGN-projection from laminae A and A1 is strong to area 17 (V1) and weaker and more variable to area 18 (V2) [63,64]. A small projection from one LGN lamina to area 19 (V3) is mentioned in only one [64] out of 5 reports (review, [50]). MIN projects mainly to V2 and V3, the geniculate wing only to V2 [64]. Orban´s review [50] clearly shows that the connections between the LGN laminae and the visual cortex in mammals are as variable as that of the thalamo-wulst connections in birds—or that the correct connectivity is not yet settled in both systems.

While all of the previous comparisons are supporting the anatomical equivalence of the visual cortex and the avian visual wulst, there is one problem, which has not been mentioned as yet. While V1, the striate cortex, is the telencephalic target of the geniculocortical pathway, the other topographically organised areas V2 to V5 are said to belong to the extrageniculocortical pathway and thus to the system which is equivalent to the tectofugal pathway in birds. If we consider the posterolateral map as equivalent to V1 and the others to, e.g., V2 and V3, the latter should receive input from the tectofugal (collothalamic) pathway. Indeed, Chaves et al. [65] showed that reversal learning of colours which had previously been shown to be affected by wulst lesions [66] is not affected by lesions of the thalamic relay nuclei, but by lesions of n. rotundus of the tectofugal pathway. They concluded that there should be connections between the tecto- and thalamofugal pathway mediating this effect. However, we were not able to find projections emanating from the entopallium or the adjacent nido-mesopallial areas, which receive entopallial efferents. Thus, the above hypothesized relation of our 3 maps with V1—V3 in mammals awaits further experimental evidence.

A more general problem is the question of whether a certain functional architecture relates to a certain behavioral function. If we assume that the visual cortex and the visual wulst are similar in their architecture, should they also serve similar behavioural functions? The answer most likely depends on how we define "behavioural function". In our case, visual wulst and visual cortex both hold an orderly representation of the visual field, and such topographic maps are most probably an ideal basis for fast feature detection (e.g. [67]) and object localization [68]. In the case of the visual wulst, we have argued that feature detection or identification may not be its predominant task because there is no foveal overrepresentation which enhances the resolution of the system and supports stimulus identification [13]. Instead, as mentioned earlier, the wulst may rather be involved in localisation tasks and/or orientation in space [69,70,71,72], probably mediated by a wulst-hippocampus connection, and may also play a role in magnetic field orientation [19,20]. Shimizu and Watanabe [1] presume that the thalamofugal system may not be involved in some sort of reflexive reaction to visual stimuli, as it has been shown for the optic tectum of the tectofugal system, where electrical stimulation of a distinct point of the topographic map leads to a head movement bringing the fovea to fixate the corresponding part of the visual field [73]. Instead, it might be involved in visually guided actions which include some planning and learning or, as Shimizu and Watanabe [1] put it, a certain degree of "cognitive flexibility". This has been shown for example by Shimizu and Hodos [66] who demonstrated that wulst lesions affect reversal learning, an operant visual task where the positive visual stimulus is changing to negative after a number of correct choices.

For the mammalian visual system, it has initially been thought that the geniculocortical system is mainly involved in identification tasks, while the extrageniculocortical projection serves for localization [74]. So far, the avian thalamofugal pathway appears not to be comparable to the geniculocortical system of mammals. With the idea of two visual streams emanating from the primary visual areas in mammals [75,76], however, the picture has changed. The dorsal of the two visual streams emanating from the primary visual cortex has been shown to be involved in localization and control of own movements in the environment, and thus be comparable in function with the thalamofugal projection in birds. However, the separation of the two visual streams occurs at the level of the visual cortex in mammals, and we have as yet no hints how information is processed when it has passed the bird visual wulst.

Taken together, the present imaging, electrophysiological and tracing results provide further evidence that the functional organisation of the avian visual wulst is comparable to that of the mammalian visual cortex. Furthermore, the new data may also give hints how to proceed to further elucidate the equivalence of avian and mammalian visual structures. Nevertheless, there are still many open questions. Given the physiological differences between the retinotopic wulst maps, it would be interesting to analyze whether they are related to different roles in behavioural guidance. For example, might it be that the posterior map is involved in spatial orientation, and the frontal map is necessary for magnetic orientation? The reverse might also be true, or the third map takes over one of the tasks. One should also not forget that the present results are just a first step towards a more detailed description of the functional architecture of the visual wulst. Closer examination of this structure will most probably not only refine our picture, but also raise new questions, and refine our current theories.
